# Adipose tissue is less responsive to food restriction anti-inflammatory effects than liver, muscle, and brain in mice

**DOI:** 10.1590/1414-431X20188150

**Published:** 2018-12-10

**Authors:** M.M. Antunes, C.B. de Almeida-Souza, G. Godoy, A.R. Crisma, L.N. Masi, R. Curi, R.B. Bazotte

**Affiliations:** 1 Departamento de Farmacologia e Terapêutica, Universidade Estadual de Maringá, Maringá, PR, Brasil; 2 Departamento de Análises Clínicas, Universidade Federal do Paraná, Curitiba, PR, Brasil; 3 Programa de Pós-Graduação Interdisciplinar em Ciências da Saúde, Universidade Cruzeiro do Sul, São Paulo, SP, Brasil

**Keywords:** Caloric restriction, Pro-inflammatory cytokines, Anti-inflammatory cytokines, Acetyl-CoA carboxylase, Disease prevention

## Abstract

High caloric intake promotes chronic inflammation, insulin resistance, and chronic diseases such as type-2 diabetes, which may be prevented by food restriction (FR). The effect of FR on expression of pro-inflammatory and anti-inflammatory genes in adipose tissue, liver, muscle, and brain was compared. Male Swiss mice were submitted to FR (FR group) or had free access to food (control group) during 56 days. The liver, gastrocnemius muscle, brain, and epididymal white adipose tissue (WAT) were collected for analysis of gene expressions. FR attenuated inflammation in the liver, brain, and gastrocnemius muscle but did not markedly change inflammatory gene expression in epididymal WAT. We concluded that adipose tissue was less responsive to FR in terms of gene expression of pro-inflammatory and anti-inflammatory genes.

## Introduction

Inflammation in the tissues has been associated with excessive caloric intake and sedentarism (1). Prolonged periods of inflammation may cause diseases such as atherosclerosis, insulin resistance, type-2 diabetes, liver steatosis, and neurological disorders. These chronic diseases exhibit marked inflammation in the liver, skeletal muscle, brain, and adipose tissue (2,3).

One effective strategy to prevent inflammation induced by high caloric diet is food restriction (FR) (4). FR has been recommended to reduce inflammation in the liver, brain, muscle, and white adipose tissue (WAT) (5,6).

In spite of this, to clarify the mechanisms involved in the prevention of inflammation by FR more studies are required. For instance, the following question remains unanswered: does attenuation of the inflammation state induced by FR vary depending on the tissue?

In order to address this question, the effect of FR on expression of pro-inflammatory and anti-inflammatory genes in the liver, gastrocnemius muscle, brain, and WAT was compared.

## Material and Methods

### Animals and treatments

Male Swiss mice (*Mus musculus*) weighing approximately 35 g were used in the experiments. The animals were housed in a room with a controlled temperature of 23°C and an automatically controlled photoperiod (12-h light/12-h dark). The Animal Ethics Committee of State University of Maringá approved the experimental protocol (1067160216/CEUA) used in accordance with the international law on protection and use of animals.

Mice were randomly divided into two groups and were allocated one per cage. The control group (C group) had free access to food (daily ingestion about 33.3 kcal), whereas the FR group received 22.8 kcal/day (31.5% lower caloric intake compared to the C group). Food was provided daily to the FR group at 9 a.m. Body weight was determined at day zero and weekly during the 56-day caloric restriction for all mice. During this period, all animals had free access to water.

All animals received a purified diet for laboratory rodents as proposed by the American Institute of Nutrition for maintenance of laboratory adult rodents (AIN-M-93). The diet composition was described in our previous publication (2).

After 56 days of caloric restriction or free-fed diets, all animals were fasted for 15 h and killed by decapitation. Blood was collected for measurements of serum glucose, triacylglycerol, and cholesterol concentrations according to the manufacturer's instructions (Labtest®, Brazil). The liver, gastrocnemius muscle, brain, and epididymal WAT were quickly and carefully removed, weighed, frozen in liquid nitrogen, and stored at –80°C until gene expression analysis was performed.

### Gene expression analysis

Expressions of F4/80, interleukin 1β (IL-1β), tumor necrosis factor alpha (TNF-α), IL-6, and IL-10 genes were evaluated in the liver, gastrocnemius muscle, brain, and epididymal WAT.

Expressions of collagen type I and acetyl-CoA carboxylase 1 (ACC1) were measured in the liver while IL-4 was measured in gastrocnemius muscle. Expressions of cyclooxygenase-2 (Cox2), inducible nitric oxide synthase (iNOS), and Ox42 Itgam were measured in the brain.

Total RNA was extracted from liver (20 mg), brain (20 mg), gastrocnemius muscle (20 mg), and epididymal WAT (100 mg) using Trizol reagent (Invitrogen Life Technologies, USA). RNA was quantified by spectrophotometry in a Nano Drop 2000 (ThermoScientific, Uniscience, Brazil) and the purity degree was determined by the 260/280 nm ratio.

Reverse transcription to cDNA was performed using the High-Capacity cDNA kit (Applied Biosystems, USA). Real-time PCR (RT-PCR) using SYBR Green as the fluorescent dye (Invitrogen Life Technologies) was used to measure gene expression. Gene expression was estimated using the comparative Ct method (Ct=threshold cycle, the cycle number at which the PCR product reaches the detection threshold), considering the expression of β2-microglobulin gene (β2m) as the standard reference gene. The relative amounts of each transcript were analyzed using the 2−ΔC(t) method. The primer sequences were: F4/80, NM_010130.4, sense CCTGAACATGCAACCTGCCAC, antisense GGGCATGAGCAGBCTGTAGGATC; Type I collagen, NM_009931.2, sense CTCTATGTCCAAGGCAACGAG, antisense TCACAAACCGCACACCTG; ACC1, NM_133360.2, sense GAGAGGGGTCAAGTCCTTCC, antisense AAAACATCCACTTCCACACACGA; IL-6, NM_001314054.1, sense GGTAGCATCCATCATTTCTTTG, antisense CGGAGAGGAGACTTCACAAGAG; IL-1β, NM_008361.4, sense GGCAGCTACCTGTGTCTTTCCC, antisense ATATGGGTCCGACAGCACGAG; TNF-α, NM_001278601.1, sense TCTTCTCATTCCTG CTTGTGGC, antisense CACTTGGTGGTTTGCTACGACG; IL-10, NM_010548.2, sense TGCCAAGCCTTATCGGAAATG, antisense AAATCGATGACAGCGCCTCAG; IL-4, NM_021283.2, sense CCATCTGTGGTGTTCTTCGTTGCTG, antisense ATCCACGGATGCGACA; Cox 2, YP_001686701.1, sense AACATCCCCTT CCTGCGAAG, antisense AAGTCCACTCCATGGCCCAG; Itgam, NM_001082960.1, sense TAATGACTCTGCGTTTGCCCTG, antisense ATTGGAGCTGCCCACAATGAG; iNOS, NM_001313921.1, sense CGGCAAACCCAAGGTCTACG, antisense CACCTGCTCCTCGCTCAAGTTC; β2M, NM_009735.3, sense AATGTGAGGCGGGTGGAACTG, antisense CATGGCTCGCTCGGTGACC.

### Statistical analysis

Data are reported as means±SE. Student's *t*-test was used to assess differences between means with the Graph-Pad Prism Version 5.0 software (GraphPad Software, USA).

## Results

Body weight gain was slower in the FR group (P<0.05). After 56 days of caloric restriction, the FR group had lower body mass and body weight gain than C group (P<0.05).

The FR had lower gastrocnemius muscle weight and epididymal WAT weight (P<0.05). However, the weight of the liver and brain were not different between the groups. Blood levels of glucose, triacylglycerol, and cholesterol were also lower in the FR group (P<0.05) (Table 1).

**Table 1:** Body weight, body weight gain, and gastrocnemius muscle, liver, brain, and epididymal white adipose tissue (WAT) weights, and blood levels of glucose, triacylglycerol, and cholesterol.

	Control group	Food restriction group
Body weight (g)	50.50±1.78	37.10±0.73*
Body weight gain (g)	19.00±1.65	5.50±0.94*
Gastrocnemius muscle weight (g)	0.47±0.01	0.41±0.01*
Gastrocnemius muscle weight (g/100 g body weight)	0.95±0.03	1.11±0.03*
Liver weight (g)	1.61±0.06	1.45±0.04
Liver weight (g/100 g body weight)	3.20±0.09	3.92±0.12*
Brain weight (g)	0.30±0.01	0.32±0.01
Brain weight/Body weight (g/100 g body weight)	0.67±0.02	0.87±0.02*
Epididymal WAT weight (g)	1.94±0.19	1.33±0.09*
Epididymal WAT weight (g/100 g body weight)	3.79±0.26	3.56±0.20
Glucose (mg/dL)	112.10±5.39	91.47±2.32*
Triacylglycerol (mg/dL)	69.55±6.11	43.11±2.54*
Cholesterol (mg/dL)	192.70±9.79	160.80±6.06*

Data are reported as means±SE (n=10). *P<0.05 compared with the Control group (Student's *t*-test for unpaired samples).

The liver relative ACC1 mRNA expressions (means±SE of 8–10 mice per group) were 3.23±1.48 for the C group and 23.25±8.23, for FR group (P<0.05).

**Figure 1:**
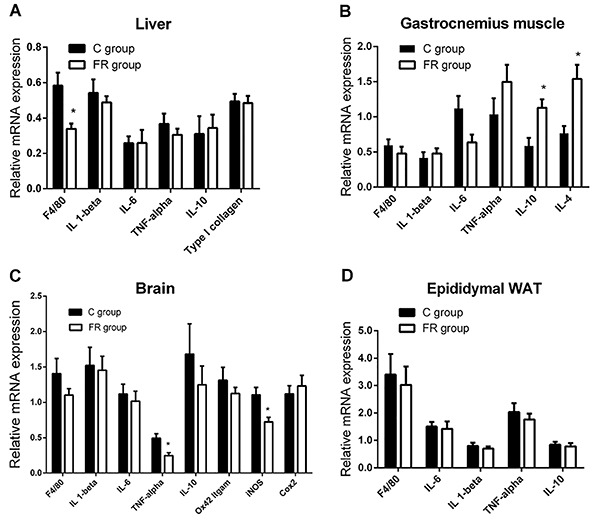
Relative mRNA expression in liver (**A**), gastrocnemius muscle (**B**), brain (**C**), and epididymal white adipose tissue (WAT) (**D**) in free-fed (C group) or food-restricted mice (FR group). Cox 2: cyclooxygenase 2; IL: interleukin; iNOS: inducible nitric oxide synthase; Ox42 itgam: integrin subunit; TNF-alpha: tumor necrosis factor-alpha. The mRNA expression was determined by RT-PCR. β2-microglobulin (β2m) was used as housekeeping gene. The relative amounts of each transcript were analyzed using the 2−ΔC(t) method. Data are reported as means±SE (n=8-10). *P<0.05 compared with the C group (Student's *t*-test for unpaired samples).

The FR group had lower (P<0.05) gene expression of F4/80 in the liver (Figure 1A) and higher (P<0.05) gene expression of IL-10 and IL-4 in the gastrocnemius muscle (Figure 1B). The brain of FR mice had lower iNOS and TNF-α gene expressions than C group (P<0.05, Figure 1C). Epididymal WAT did not exhibit any difference between groups for all genes evaluated (Figure 1D).

## Discussion

FR attenuated inflammation in the liver, brain, and gastrocnemius muscle but did not markedly change inflammatory gene expression in epididymal WAT.

Liver ACC1 expression was seven-fold higher in the FR group. The higher expression of ACC1 is a metabolic adaptation and indicates enhanced capacity of *de novo* synthesis of fatty acids and increased potential for lipid accumulation during food restriction (7,8).

F4/80, IL-1β, IL-6, TNF-α, and IL-10 gene expression were analyzed in all tissues for two reasons. First, we previously reported changes in gene expression of these five genes in the liver of C group indicating an inflammatory state (2). Second, F4/80, IL-1β, IL-6, and TNF-α are biomarkers of inflammatory states (1–9), while IL-10 represents one of the major anti-inflammatory cytokines (10). Moreover, these cytokines have profound metabolic effects creating complex networks of interactions between cells and tissues, leading to multiple inflammatory cascades (1–6). Additional specific genes better represent inflammation in different tissues. In the liver, for example, type I collagen indicates fibrosis and chronic liver injury (3). In the brain, Ox42 itgam, iNOS, and Cox2 genes are well established inflammatory biomarkers (11,12), and IL-10 and IL-4 have a pivotal role as anti-inflammatory cytokines (10).

The liver from FR mice exhibited decreased F4/80 expression, which is one of the most specific cell-surface markers of macrophage and thus, a marker of inflammation (2). The lower expression of F4/80 in the FR group indicated less macrophage infiltration in the liver and therefore attenuation of inflammation and fatty liver disease (9).

Gastrocnemius muscle exhibited increased expressions of anti-inflammatory cytokines IL-10 and IL-4 due to FR. IL-10 is an important regulator of IL-6, TNF-α, and IL-1β levels whereas IL-4 plays an important role in skeletal muscle regeneration (10).

FR prevents brain diseases (11). This beneficial effect of FR includes metabolic reprogramming and changes in the levels of cytokines that regulate neuroinflammation (12). In agreement with this statement, we observed lower brain iNOS and TNF-α expressions.

In contrast with other tissues, epididymal WAT did not exhibit any difference in the expressions of the genes measured. The absence of changes in inflammatory gene expressions in WAT reported in this study, however, does not discard the possibility that differences may occur with prolonged periods of FR or other types of diets. For example, nine-month FR, as well high-fat diet models associated with FR in mice down-regulates expressions of inflammatory genes in WAT (13,14).

Taken together, our results and findings reported by others (13–15) suggest that the attenuation of the inflammation state induced by FR not only is time-dependent but also varies with the tissue.

The clinical relevance of this investigation is that the FR anti-inflammatory effects could be progressive, and prolonged periods might be necessary to reach the maximum benefits of food restriction.
